# Chalcones, stilbenes and ketones have anti-infective properties via inhibition of bacterial drug-efflux and consequential synergism with antimicrobial agents

**DOI:** 10.1099/acmi.0.000105

**Published:** 2020-02-18

**Authors:** Lauren Hellewell, Sanjib Bhakta

**Affiliations:** ^1^​ Division of Biosciences, Institue of Structual and Molecular Biology, University College London, London, WC1E 6PT, UK; ^2^​ Department of Biological Sciences, Institute of Structural and Molecular Biology, Birkbeck, University of London, London, WC1E 7HX, UK

**Keywords:** antimicrobial resistance, efflux pumps, synergism, chalcone, stilbene, ketone, whole-cell phenotypic evaluation, new drug discovery

## Abstract

With antimicrobial resistance creating a major public health crisis, the designing of novel antimicrobial compounds that effectively combat bacterial infection is becoming increasingly critical. Interdisciplinary approaches integrate the best features of whole-cell phenotypic evaluation to validate novel therapeutic targets and discover new leads to combat antimicrobial resistance. In this project, whole-cell phenotypic evaluation such as testing inhibitors on bacterial growth, viability, efflux pump, biofilm formation and their interaction with other drugs were performed on a panel of Gram-positive, Gram-negative and acid-fast group of bacterial species. This enabled additional antimicrobial activities of compounds belonging to the flavonoid family including ketones, chalcones and stilbenes, to be identified. Flavonoids have received renewed attention in literature over the past decade, and a variety of beneficial effects of these compounds have been illuminated, including anti-cancer, anti-inflammatory, anti-tumour as well as anti-fungal and anti-bacterial. However, their mechanisms of action are yet to be identified. In this paper, we found that the compounds belonging to the flavonoid family exerted a range of anti-infective properties being identified as novel efflux pump inhibitors, whilst offering the opportunity to be used in combination therapy. The compound 2-phenylacetophenone displayed broad-spectrum efflux pump inhibition activity, whilst trans-chalcone, displayed potent activity against Gram-negative and mycobacterial efflux pumps causing inhibition higher than known potent efflux pump inhibitors, verapamil and chlorpromazine. Drug-drug interaction studies also highlighted that 2-phenylacetophenone not only has the potential to work additively with known antibacterial agents that affect the cell-wall and DNA replication but also trans-chalcone has the potential to work synergistically with anti-tubercular agents. Overall, this paper shows how whole-cell phenotypic analysis allows for the discovery of new antimicrobial agents and their consequent mode of action whilst offering the opportunity for compounds to be repurposed, in order to contribute in the fight against antimicrobial resistance.

## Impact statement

This whole-cell phenotypic evaluation-based research project aims to exploit a group of compounds for the early-stage drug discovery against a selected panel of WHO priority bacterial pathogens. Our goal was to identify ‘hits’ which would be worthy of further optimisation as adjuvants for existing antibiotic therapy. This project has provided the first step in addressing an important issue, i.e. the urgent need for alternative approaches to combat antimicrobial drug resistance (AMR).

##  Introduction

Bacterial resistance is a serious problem that is a well-established public health concern [1]. Most of the antibiotics that are available in the market today were discovered in the golden antibiotic era; in the period from 1940’s to 1960’s [2]. It was a very successful period for discovering different classes of antibiotics; mostly antibiotics from natural sources, and also some synthetic analogues [3]. Most importantly, this period marked progress in medical microbiology that heightened our understanding of the bacteria itself and the molecular mechanisms of antibiotics. However, since then, only four new classes of antibiotics have been approved for sale [4]. Pathogens have since developed a number of mechanisms which render said antibiotics unusable against certain bacterial infections [5]. Consequently, the majority of the new drugs currently in clinical settings are derivatives of the chemical classes of known antibiotics and are already met with underlying antibiotic resistance mechanisms [6]. Although there are strategies in place for controlling the expansion of antibiotic resistance, including the prevention of the misuse of antibiotics [7], other novel tactics need to be brought forward, such as the development of new infectives from brand new chemical classes. Therefore, how do we accelerate the discovery pipeline of new drugs with novel mechanisms of antibiotic action? [8, 9] Drug discovery via whole-cell phenotypic evaluation has been the most successful method for discovering antibiotics with a novel chemical class such as cephalosporin, rifampicin and vancomycin. The Waksman platform is a well-known example of whole cell screening platform that was used to screen soil-derived *Streptomycetes* for antibacterial activity which has successfully discovered antibiotics such as streptomycin and neomycin [10]. In addition, emerging methods using transcript quantification, public databases, combined with machine-learning tools, are providing ground-breaking new and alternative screening strategies by augmenting phenotypic screening results [11]. Whole-cell evaluation offers a number of advantages over other drug discovery methods which includes the evaluation of a compounds’ efficiency in penetrating bacterial cell-walls and for discovering novel efflux inhibitors [12]. In addition, whole-cell evaluation is a sensible platform combined with target drug design for the discovery of compounds that are prodrug which require intracellular metabolism to become active [13]. Alternative platforms primarily focusing on specific biomolecule targets, disregarding whole-cell screening, have therefore faced several challenges in new antibiotic development. Lastly, whole-cell phenotypic screening also allows for identification of various other mechanisms of action known antimicrobial agents could have. Many biologically active compounds have been shown to have more than one endogenous mechanism of action. This is termed by ‘pleiotropy’ which looks into the ability of the biologically active compounds to implement more than one mechanism of action [14]. With this knowledge, known and putative antimicrobial compounds belonging to the flavonoid family [15–19], were investigated in this study to evaluate their whole-cell phenotypic effect, with the aim to fill the existing research gap of identifying any additional mechanisms of action the compounds may possess.

## Materials

### Bacterial strains

*Escherichia coli* K12 (* E. coli *) strain, *Bacillus subtilis* strain 168 (* B. subtilis *), *Mycobacterium smegmatis* mc^2^155 ATCC700084 (* M. smegmatis *), *Mycobacterium aurum* ATC:NC 10437 (* M. aurum *) and *Mycobacterium bovis* BCG Pasteur ATCC 35734 (* M. bovis* BCG), purchased from UK National Collection of Type Cultures (NCTC) were used as a representative for Gram-negative, Gram-positive and mycobacterium species.

### Compound library

The compound library used in this study included; trimethoprim, methotrexate, pyrimethamine, 2-chloro-4,6-diamino-1,3,5-triazine, 2-phenylacetophenone, trans-chalcone, trans-stilbene, trans-stilbene oxide, luteolin, 2,4-diaminoquinazine and triamterene, Table 1.

**Table 1.** The compound library used in this study. MW; molecular weight, HBD; hydrogen bond donors, HBA; hydrogen bond acceptors, LogP; partition coefficient. Compounds in this library fall into categories; diaminopyrimidines, diaminopteridines, diaminotriazines, diaminoquinazolines, flavonoids, stilbenes and deoxybenzoins. All compounds in this library adhere to Lipinski's rule of five

Drug	Structure	Formula	Properties
**Trimethoprim** (**Compound 1/C1**)	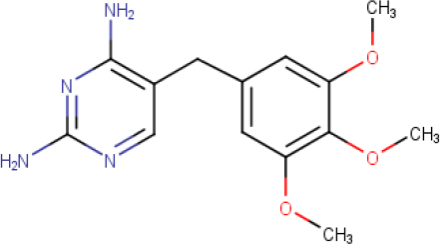	C_14_H_18_N_4_O_3_	Form: Powder MW: 290.32 g/mol HBD: 2 HBA: 7 LogP: 0.9 Solvent: DMSO
**Methotrexate** (**Compound 2/C2**)	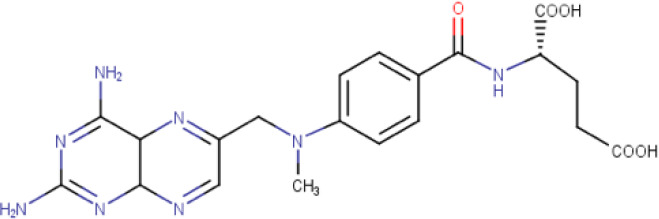	C_20_H_22_N_8_O_5_	Form: Yellow powder MW: 454.44 g/mol HBD: 5 HBA: 12 LogP: −1.9 Solvent: DMSO
**Pyrimethamine** (**Compound 3/C3**)	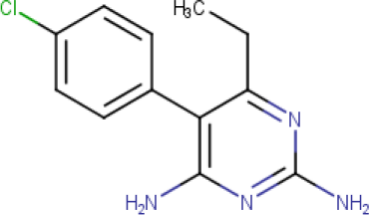	C_12_H_13_CIN_4_	Form: Solid MW: 248.72 g/mol HBD: 2 HBA: 4 LogP: 2.7 Solvent: DMSO
**2-chloro- 4,6 -diamino- 1,3,5 triazine** (**Compound 4/C4**)	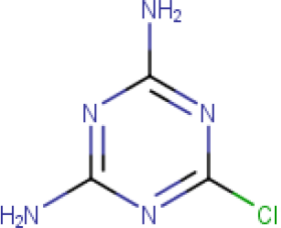	C_3_H_4_CIN_5_	Form: Powder MW: 145.55 g/mol HBD: 2 HBA: 3 LogP: 0.25 Solvent: DMSO
**2-phenylacetophenone** (**Compound 5/C5**)	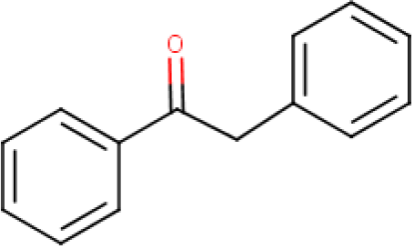	C_14_H_12_O	Form: light yellow crystalline powder MW: 196.249 g/mol HBD: 0 HBA: 1 LogP: 3.2 Solvent: DMSO
**Trans-chalcone** (**Compound 6/C6**)	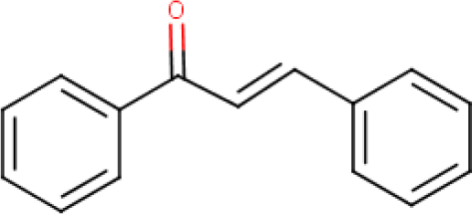	C_15_H_12_O	Form: Powder MW: 208.26 g/mol Solvent: DMSO HBD: 0 HBA: 1 LogP: 4.0
**Trans-stilbene** (**Compound 7/C7**)	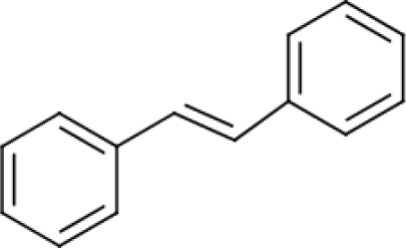	C_14_H_12_	Form: White crystalline powder MW: 180.25 g/mol Solvent: DMSO HBD: 0 HBA: 0 LogP: 4.8
**Trans-stilbene oxide** (**Compound 8/C8**)	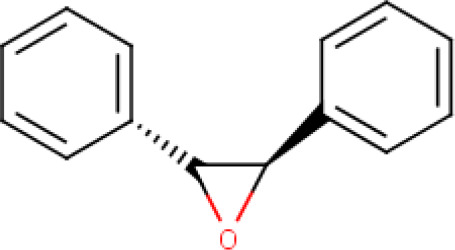	C_14_H_12_O	Form: White crystalline powder MW: 196.24 g/mol Solvent: DMSO HBD: 0 HBA: 1 LogP: 3.5
**Luteolin** (**Compound 9/C9**)	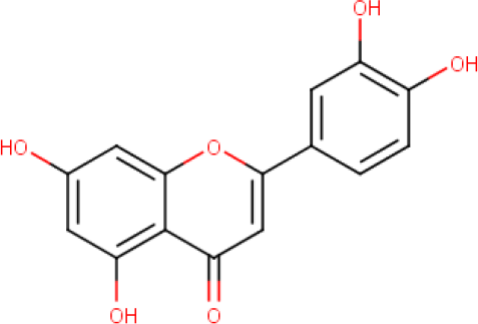	C_15_H_10_O_6_	Form: Yellow powder MW: 286.24 g/mol Solvent: DMSO HBD: 4 HBA: 6 LogP: 2.5
**2,4- diaminoquinazine** (**Compound 10/C10**)	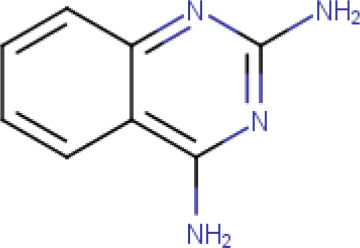	C_8_H_8_N_4_	Form: White powder MW: 160.18 g/mol Solvent: DMSO HBD: 2 HBA: 4 LogP: 1
**Triamterene** (**Compound 11/C11**)	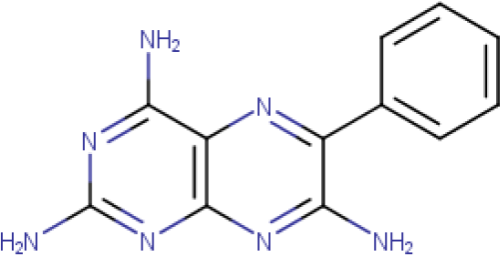	C_12_H_11_N_7_	Form: Yellow powder MW: 253.269 g/mol Solvent: Formic acid HBD: 3 HBA: 7 LogP: 0.98

The controls (existing antibiotics) used in this study included; ampicillin, kanamycin, isoniazid, ethambutol and rifampicin.

## Methods

### Resazurin microtiter plate assay (REMA)

The inhibitor compounds were further tested using a whole-cell phenotypic screen, REMA following modified [20, 21]. In brief an additional fluorescence reading step was included to optimise the method. This enabled elimination of any compound and dye interference. The reading of the florescence was done using a plate reader (FLUOstar OPTIMA, BMG Labtech, UK) with the settings as follows; 544 nm and 590 nm for excitation and detection of fluorescence (emission), gain 2200 and a temperature of 37 °C. The MIC was determined as the lowest concentration of the drug at which no growth of bacteria was observed. The experiment was performed in triplicate.

### Cytotoxicity and selectivity index

For the evaluation of the cytotoxicity profiles an established laboratory protocol was followed. In brief, the cytotoxicity assay was performed in a 96-well plate. Two-fold serial dilutions were made by transferring 100 µl from the first row to the next row containing 100 µl of complete RPMI media. This step was repeated until the second last row as the last row was kept as a control in which no compound was added (media only). Each compound was tested in biological triplicates, using three separate cell culture lines. In each well, 100 µl of cell line (RAW264.7 or THP1), which had been passaged and normalised to a concentration of 5×10^5^ cells ml^−1^, was added. The plate was then incubated in a CO_2_ incubator. Following a 48 h incubation, the plate was washed with 1XPBS by pipetting and fresh complete RPMI was added to each well. Then 30 µl of freshly prepared resazurin solution at 0.01 % was added to the media and this plate was then incubated overnight in the CO_2_ incubator. The following day, the colour change was observed, and fluorescence intensity was measured using FLUOstar OPTIMA microplate reader at λexc=540 nm, λemi=590 nm. The growth inhibitory concentration was determined based on a resazurin fluorescence assay. The 50 % growth inhibitory concentration (GIC_50_) is the concentration which gave fluorescence at the midpoint of the highest fluorescence detected and the lowest fluorescence.

The selectivity index/antibacterial selectivity was determined as the ratio between the GIC_50_ on macrophage (RAW 264.7 and THP1) and the MIC observed in the REMA assays by using the formula: SI=GIC µg ml^−1^/MIC µg ml^–1^.

### Efflux pump analysis

The EtBr efflux assay to test the inhibitor compounds (at ¼ × MIC) was performed in a 96-well flat bottom plate (Greiner Bio-One). Meanwhile for controls using known efflux inhibitor chlorpromazine and verapamil had a final concentration of ½ × MIC for each organism used.

The method was performed using the standard laboratory protocol. Bacterium cultures; *E. coli *, *B. subtilis *, *M. smegmatis, M. aurum* and *M. bovis* BCG were passaged at least twice, and grown over night until the OD_600_ reached 0.8. The OD was adjusted to 0.4 by taking equal volumes of the culture and M7H9 (5 ml of each), making sure the final volume of the suspension remained at 10 ml. The cells were collected by centrifugation at a speed of 3000 ***g*** for 10 min after which the supernatant was removed by decanting. The pellet was resuspended with in 10 ml of sterile 1 × PSB, using a vortex to distribute the cells uniformly.

The test samples contained 500 µl of culture resuspended in 1 × PBS at OD 0.4, 2.5 µl of 80 % glucose (to keep the cells viable and metabolically active), 1.25 µg ml^−1^ EtBr (as the substrate for the efflux pumps) and the compounds being tested were at ¼ × MIC concentrations. Blank samples contained all the components mentioned above except the bacterial suspension, which was replaced with 1 × PBS, required to correct the intrinsic florescence of the inhibitors. All the test samples and blanks were prepared in triplicate. The continuous reading of the florescence was done using a plate reader (FLUOstar OPTIMA, BMG Labtech, UK) with the settings as follows; 540 nm and 590 nm for excitation and detection of fluorescence (emission), gain 2200, a temperature of 37 °C and a cycle of measurement every 60 s for a total period of 60 min.

### Biofilm inhibition analysis

Biofilm growth was started by thawing cryopreserved bacterial cell culture, *M. smegmatis *, followed by passaging three times in M7H9 supplemented with ADC and grown until OD reached the upper mid log phase (OD 2.0). This is when molecules within the bacterial population switch on a communication system known as quorum sensing. This culture was therefore used and diluted 1 : 100 times in Sauton’s media. This included triplicates of each concentration for each compound along-side triplicates of a positive control (diluted cell culture in Sauton’s media), a 1 % DMSO control (M7H9 supplemented with ADC, diluted cell culture and 20 ul of DMSO) and a negative control (M7H9 supplemented with ADC alone). Then 2 ml of the diluted cell culture was added to each Eppendorf tube along with specific volumes of each compound to make up certain concentration ranges. Three compounds were tested at varying concentrations; trans-chalcone 500–31.25 µg ml^−1^, 2-phenylacetophenone 1000–125 µg ml^−1^ and trans-stilbene oxide 1000–125 µg ml^−1^. The plastic test tubes were then incubated for 5 days at 35 ˚C, then biofilm formation was then examined for direct, visible phenotypic changes to the biofilm.

### Drug-drug interaction

Compounds showing potent efflux pump inhibition on each Gram-positive, Gram-negative and mycobacterium models used in this study, were used in combination with clinically relevant compounds. Synergy testing was performed using the checkerboard assay [22–25]. This was used to determine the interaction and potency of two test articles when used concurrently. This method determined the effect on potency of the combination of antibiotics in comparison to their individual activities. This comparison was then represented as the Fractional Inhibitory Concentration (FIC) index value.

To quantify the interactions between the antimicrobial and efflux inhibitors being tested, the FIC index (the combination of antibiotics that produced the greatest change from the individual antimicrobials MIC) value is calculated for each strain and antibiotic combination: A/MIC_A_+B/MIC_B_=FIC_A_+ FI_CB_=FIC index, where A and B are the MIC of each antimicrobial in combination (in a single well), and MIC_A_ and MIC_B_ are the MIC of each drug individually. This FIC index value was then used to categorise the interaction of the two antibiotics tested [26].

## Results

### Minimum inhibitory concentration values

The minimum inhibitory concentration values displayed for the known compounds; trimethoprim, methotrexate and pyrimethamine when tested against the panel of bacterium varied from <0.98 to >500 µg ml^−1^. This was in line with what had previously been reported using various independent screening techniques. The other selected chemical entities (chalcones, stilbenes and ketones) within this study also displayed poor growth inhibitory activities when tested on each bacterial model, except trans-chalcone which was found to be selectively potent against slow-growing mycobacteria. Triamterene was abandoned from the study at this point as its dissolvent, formic acid, was causing bacterial growth inhibition instead of the compound itself, Table 2.

**Table 2.** MIC values of compounds of interest investigated using REMA analysis; against *E. coli* K12, *B. subtilis* strain 168, *M. smegmatis* mc^2^ 155*, M. aurum* ATC: NC 10437 and *M. bovis* BCG. ‘–‘ represents ‘not determined’; ‘*’ represents ‘abandoned due to insolubility’

Compound			MIC (µg/mL)		
	* E. coli *	* B. subtilis *	* M. smegmatis *	* M. aurum *	* M. bovis* BCG
**Trimethoprim**	<0.98	3.91	7.81	62.5	500
**Methotrexate**	>500	500	500	250	125
**Pyrimethamine**	25	12.5	3.13	3.13	50
**2-chloro-4,6-diamino-1,3,5-triazine**	>500	>500	>500	500	250
**2-phenylacetophenone**	>500	500	500	500	250
**Trans-chalcone**	>500	>500	125	62.5	15.60
**Trans-stilbene**	>500	>500	>500	>500	>500
**Trans-stilbene oxide**	>500	>500	>500	>500	250
**Luteolin**	>500	>500	>500	>500	>500
**2,4-diaminoquinazoline**	62.50	250	250	250	250
**Triamterene**	*	*	*	*	*
**Isoniazid**	–	–	15.60	<0.98	<0.98
**Ampicillin**	1.95	<0.98	–	–	–
**Kanamycin**	15.63	7.81	–	–	–

### Cytotoxicity and therapeutic potential evaluated

All test compounds investigated in this study were found poor in terms of their bacterial growth inhibitory properties and revealed a low selectivity index (SI) when tested against both THP-1 and RAW 264.7 cell lines. From these results the GIC_50_ and selectivity index for each compound was established, Tables 3 and 4. As expected, trimethoprim, methotrexate and pyrimethamine had high SI scores for all bacterium used in this study. This correlates with previously published results and were already on clinical trial.

**Table 3.** GIC_50_ scores

Compound	RAW 264.7 Marine Macrophage Cell Line	THP-1 Mammalian Macrophage Cell Line
	GIC_50_ (µg/ml)	GIC_50_ (µg/ml)
**Trimethoprim**	500	>500
**Methotrexate**	>500	>500
**Pyrimethamine**	>100	>100
**2-chloro-4,6-diamino-1,3,5-triazine**	125	>500
**2-phenylacetophenone**	125	250
**Trans-chalcone**	7.81	15.63
**Trans-stilbene**	>500	>500
**Trans-stilbene oxide**	125	500
**Luteolin**	31.25	>500
**2,4-diaminoquinazoline**	125	125

**Table 4.** Selectivity index (SI) of each compound. RAW 264.7 and THP-1 cell line selectivity index values are displayed below for each bacterium for the two cell lines used against a representative of Gram-ve, *E. coli* (*Ec*); Gram +ve, *B. subtilis* (*Bs*) and Acid-fast bacilli, *M. bovis* BCG (*Mb*)

Compounds	SI (RAW 264.7 Cell Line)			SI (THP-1 Cell Line)		
	*Ec*	*Bs*	*Mb*	*Ec*	*Bs*	*Mb*
**Trimethoprim**	510	510	1	510	510	1
**Methotrexate**	1	4	4	1	4	4
**Pyrimethamine**	4	16	2	4	16	2
**2-chloro-4,6-diamino-1,3,5-triazine**	0.25	0.25	0.5	1	1	2
**2-phenylacetophenone**	0.25	1	0.5	0.5	2	1
**Trans-chalcone**	0.02	0.02	0.5	0.03	0.03	1
**Trans-stilbene**	1	1	1	1	1	1
**Trans-stilbene oxide**	0.25	0.25	2	1	1	2
**Luteolin**	0.06	0.06	0.06	1	1	1
**2,4-diaminoquinazoline**	2	0.5	0.5	2	2	0.5

### Identification of novel efflux pump inhibitors

**Fig. 1.**
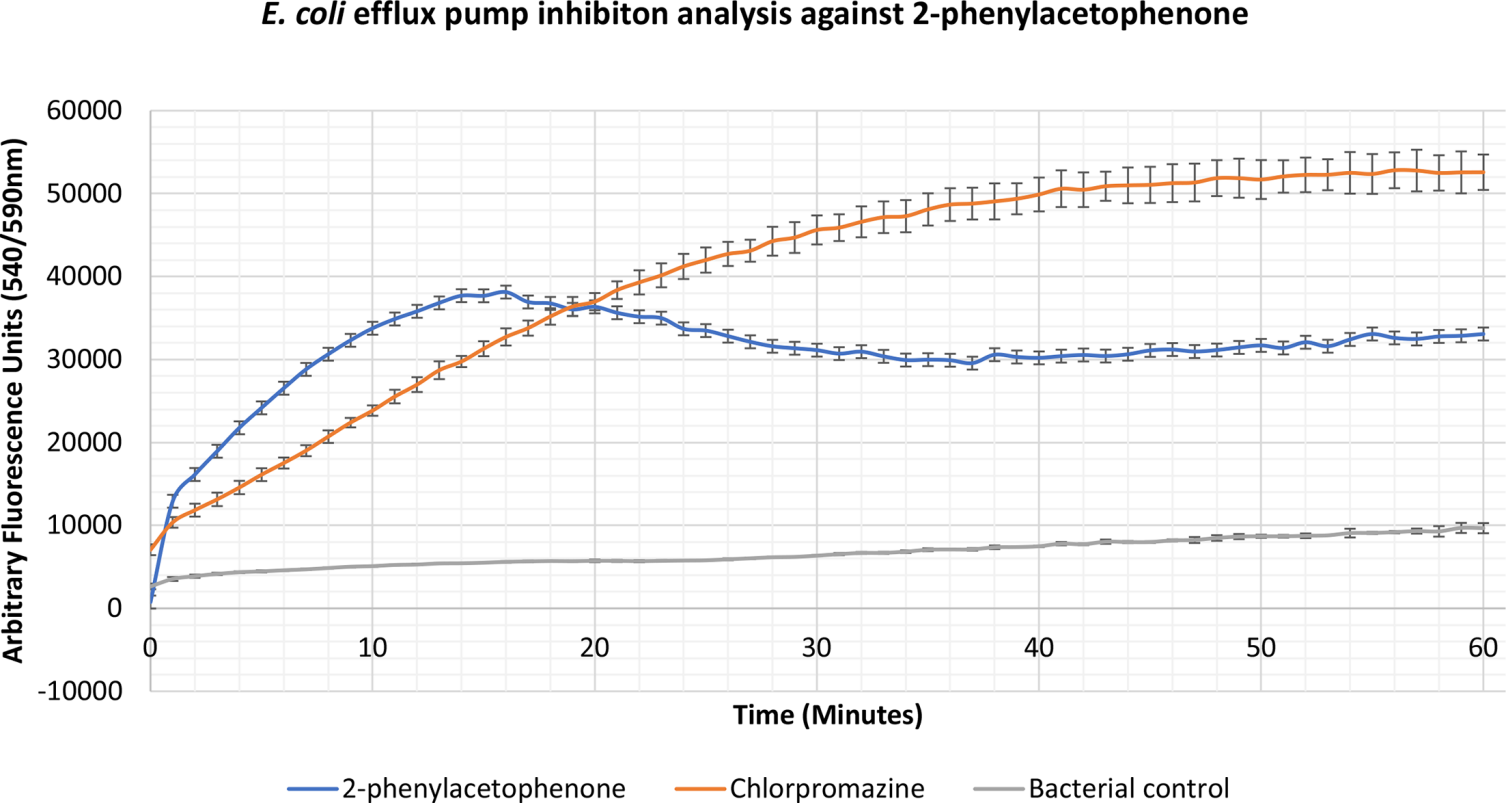
Accumulation assay using EtBr with *E. coli* K12. The bacterial control consisted of *E. coli* K12 cells with growth supplements and ethidium bromide (EtBr).

**Fig. 2.**
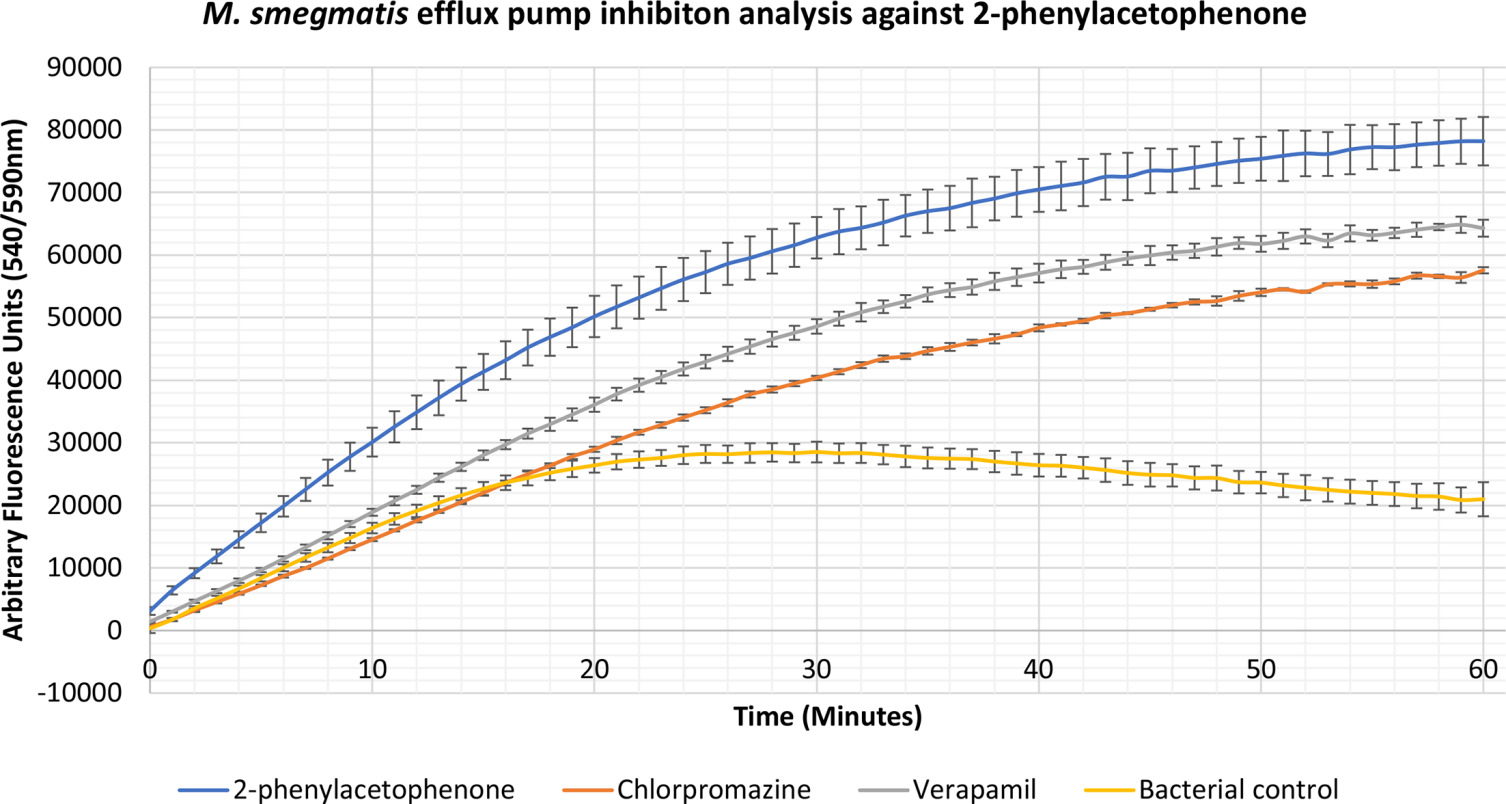
Accumulation assay using EtBr with *M. smegmatis* mc^2^ 155. The bacterial control consisted of *M. smegmatis* mc^2^ 155 cells with growth supplements and ethidium bromide (EtBr).

**Fig. 3.**
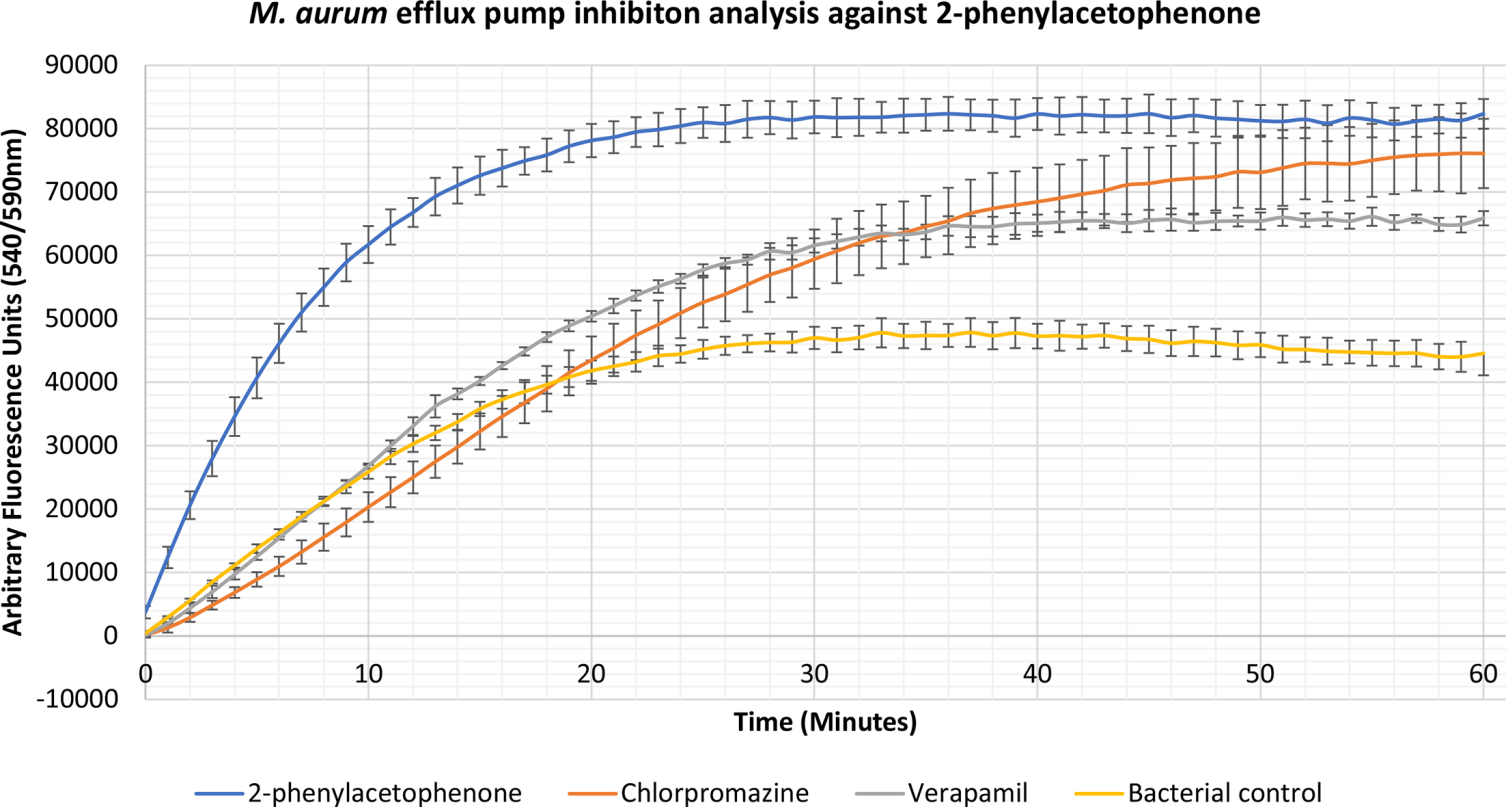
Accumulation assay using EtBr with *M. aurum* ATC: NC 10437. The bacterial control consisted of *M. aurum* ATC: NC 10437 cells with growth supplements and ethidium bromide (EtBr).

**Fig. 4.**
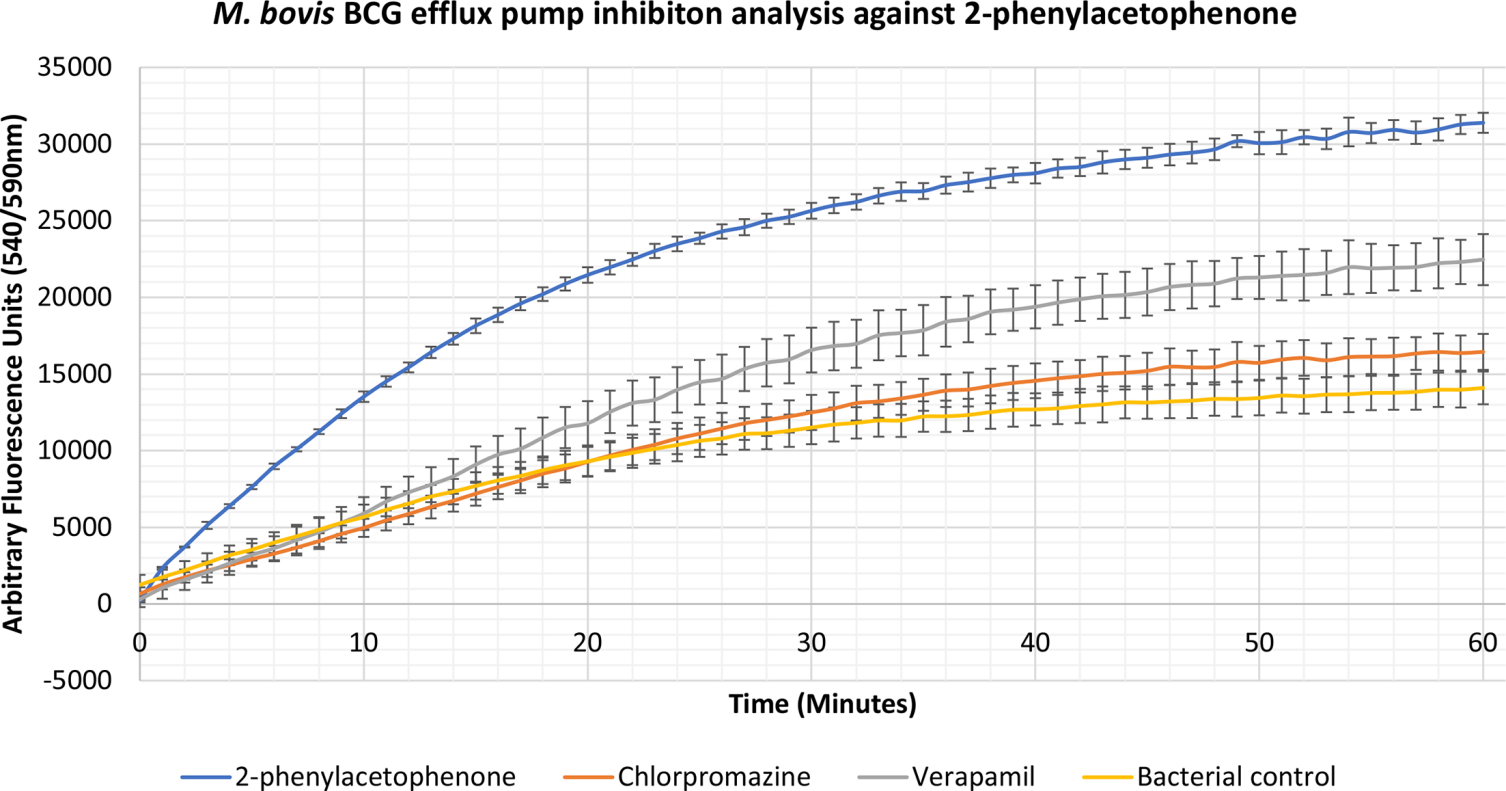
Accumulation assay using EtBr with *M. bovis* BCG. The bacterial control consisted of *M. bovis* BCG Pasteur cells with growth supplements and ethidium bromide (EtBr).

**Fig. 5.**
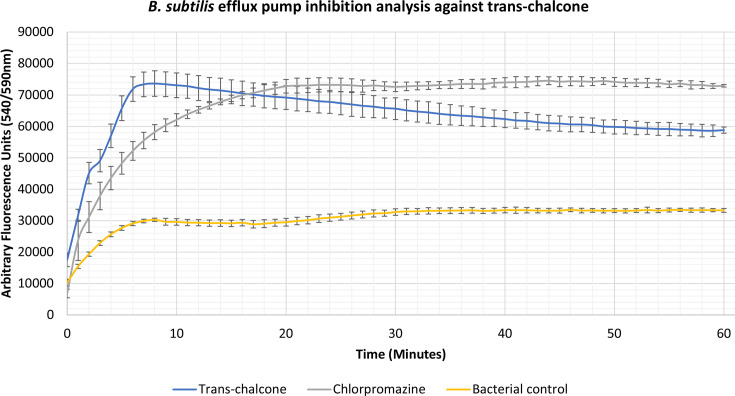
Accumulation assay using EtBr with *B. subtilis* Strain 168. The bacterial control consisted of *B. subtilis* Strain 168 cells with growth supplements and ethidium bromide (EtBr).

Compounds 2-phenylacetophenone, trans-chalcone, trans-stilbene and trans-stilbene oxide were found to inhibit the accumulation assay at ¼ MIC for a variety of Gram-positive, Gram-negative and mycobacterial species, having a better effect than two known efflux pump inhibitors, verapamil and chlorpromazine. From these results a lead compound was established which displayed the highest inhibitory activity for each bacterium. The 2-phenylacetophenone displayed broad-spectrum activity causing high inhibitory readings for the Gram-negative representative, *E. coli *, as well as the mycobacterium species; *M. smegmatis *, *M. aurum* and *M. bovis* BCG. Whilst 2-phenylacetophenone matched the efflux pump inhibition activity of known control chlorpromazine when tested against *E. coli* in the first 30 min, the compound displayed significantly higher efflux inhibitory activity compared to controls chlorpromazine and verapamil when used against the mycobacterium species (Figs 1–4). Trans-chalcone displayed high efflux pump inhibition within the first 30 min of analysis against *B. subtilis* which matched known control chlorpromazine, Fig. 5. All other compounds displayed negative results, no efflux pump inhibition matching the bacterial control or causing little efflux pump inhibition.

### Selected inhibitors ablated mycobacterial biofilm formation

**Fig. 6.**
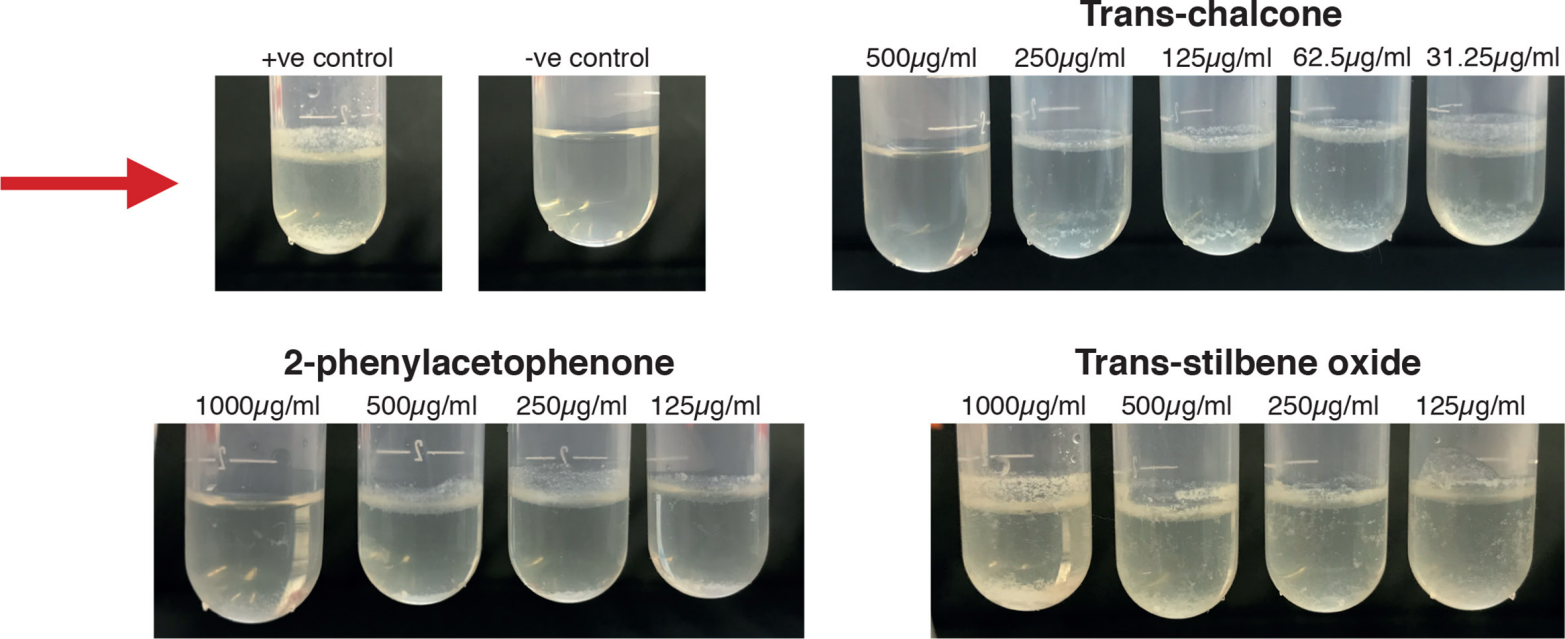
Biofilm inhibition study using *M. smegmatis* mc^2^ 155 cells against trans-chalcone, 2-phenylacephenone and trans-stilbene oxide. Positive control (+ve); *M. smegmatis* cells and growth media alone, negative control (-ve); growth media alone. The red arrow indicates biofilm formation.

Significant inhibition of biofilm formation for *M. smegmatis* was seen at 2× MIC of log phase cells for 2-phenylacetophenone and trans-chalcone. Lower concentrations of each compound did not change cell viability, but physiological changes were observed, indicating that some endogenous mechanisms were influenced, Fig. 6.

### Potent novel efflux pump and biofilm formation inhibitors displayed synergistic properties

Compounds showing significant efflux pump activity were analysed for their synergistic activities. The compound 2-phenylacetophenone displayed potent efflux pump activity for *E. coli* and therefore was tested for interactions with kanamycin and ampicillin. Potent activity for this compound was also seen when used against mycobacterial models in this study and therefore was used in combination with ethambutol, isoniazid and rifampicin against *M. smegmatis *. Trans-chalcone displayed potent efflux activity which was also seen across all bacterial models used in this study and was used in combination with kanamycin and ampicillin against *E. coli* and *B. subtilis* as well as with ethambutol, isoniazid and rifampicin against *M. smegmatis *. Trans-stilbene showed potent activity against *B. subtilis* and so was used in combination with kanamycin and ampicillin to measure their interaction. Lastly, trans-stilbene oxide displayed potent efflux pump activity against mycobacterium models used in this study and so was used in combination with ethambutol, isoniazid and rifampicin against *M. smegmatis *. The FIC index value was used to interpret what effect each compound combination was having on each bacterium; a value ≤0.5 displayed synergistic properties, >displayed additive properties whilst values of 1–4 and >4 were shown to have indifferent and antagonistic properties, Table 5. The 2-phenylacetophenone had additive effects when used in conjunction with kanamycin as well as ampicillin when used against *E. coli *. Trans-chalcone and trans-stilbene, however, had an antagonistic effect when used in conjunction with kanamycin and ampicillin against *B. subtilis *. Also 2-phenylacetophenone had synergistic effects when used with ethambutol and isoniazid against *M. smegmatis *, whilst having additive properties when used in conjunction with rifampicin. Trans-stilbene oxide, however, displayed either indifferent or antagonistic results when used in conjunction with anti-mycobacterial agents against *M. smegmatis *. To our interest, trans-chalcone displayed synergistic properties when used with all clinically relevant anti-mycobacterial agents used in this study, ethambutol, isoniazid and rifampicin when used against *M. smegmatis *.

**Table 5.** Synergy analysis with compounds showing potent efflux pump inhibition activity and known antibiotics. The FIC index (the combination of antibiotics that produced the greatest change from the individual antimicrobials MIC) value is calculated for each strain and antibiotic combination: A/MIC_A_+B/MIC_B_=FIC_A_+ FI_CB_=FIC Index. This FIC Index value was then used to categorise the interaction of the two antibiotics tested. *Ec; E. coli, Bs; B. subtilis, Ms; M. smegmatis*

Clinically relevant compounds	Putative inhibitors	FIC Index Value		
		*Ec*	*Bs*	*Ms*
**Kanamycin**	2-phenylacetophenone	1.00	–	–
	Trans-chalcone	–	>4	–
	Trans-stilbene	–	>4	–
**Ampicillin**	2-phenylacetophenone	0.99	–	–
	Trans-chalcone	–	>4	–
	Trans-stilbene	–	>4	–
**Ethambutol**	2-phenylacetophenone	–	–	0.38
	Trans-chalcone	–	–	0.38
	Trans-stilbene oxide	–	–	>4
**Isoniazid**	2-phenylacetophenone	–	–	0.26
	Trans-chalcone	–	–	0.5
	Trans-stilbene oxide	–	–	>4
**Rifampicin**	2-phenylacetophenone	–	–	0.56
	Trans-chalcone	–	–	0.38
	Trans-stilbene oxide	–	–	4.00

## Discussion

The overall aim of this study was to evaluate compounds using whole-cell phenotypic assays in order to establish any other possible mechanisms of action known and putative inhibitory compounds exhibited on a panel of Gram-positive, Gram-negative and mycobacterial species. Whilst discoveries on the molecular basis of disease provides numerous pathways in identifying new antibiotics, developing completely new antimicrobial compounds takes a significant amount of effort, money and time. Therefore, it is critical to improve these strategies to reduce both the time frame and costs of drug discovery, with the answer being drug repurposing. The use of unsuitable models and methodologies often leads to the disregard of molecules from drug development pipelines in spite of these molecules harbouring important/relevant properties that may not necessarily be anti-bacterial but may have the capacity to potentiate current treatment/reverse resistance. Therefore, it is essential that in parallel to extracting and synthesising novel chemical entities, methodologies for testing these molecules are optimised to glean maximum information on their effects against microbes, making sure that all available avenues are explored to pump-prime new antibiotic discovery, development and drug-repositioning [27].

Efflux pumps play an important role in antibiotic resistance as well as in biofilm formation [28, 29]. Consequently, results in this paper are significant in the fight against antibiotic resistance, displaying various chemical classes such as; chalcones, ketones and stilbenes, to have potential activity against such mechanisms. These are classes which have not been previously identified as efflux pump inhibitors. From efflux pump inhibition combined with synergism analysis it can be deduced that 2-phenylacetophenone has the potential to work synergistically/additively with known antibacterial agents that affect the cell wall and DNA replication across Gram-negative and mycobacterial species. Whilst trans-chalcone has the potential to work synergistically with mycobacterial agents. These results ultimately display how deoxybenzoins derivatives and chalcone chemical classes have the potential to work in combination with various known antimicrobial agents to enhance treatment and help eradicate antibacterial resistance which has not previously been looked into. However, further validation would be needed across a wider range of bacterial species, or in combination with other antibacterial agents. Another significant result includes the identification of new compounds that inhibit biofilm development could enable decreasing tuberculosis treatment that further will have a global impact in reduction of antibiotic resistance due to compliance with TB long term treatment [30, 31]. To enhance the validity of the biofilm method, it would be more appropriate to compare the MIC of stationary phase cells to biofilm MIC values. This is because as stationary phase cells are more drug tolerant for many reasons, such as having a slow metabolism, the MIC of stationary phase cells would differ from that of logarithmic phase cells. When intertwined with literature evidence that biofilms originate from a stationary population, it would therefore be more appropriate to compare MIC values between stationary phase cells and biofilms than logarithmic phase cells [32–34]. To further validation of the biofilm assay, future work would include biochemical analysis of the biofilm formation. Looking into the chemical nature of the biofilm would indicate the changing properties of the biofilm and possibly reveal which endogenous mechanism is being influenced by the specific compounds mentioned in this paper.

Unfortunately, all putative compounds looked into in this study displayed higher toxicity results. However, this was an expected result for many chemical classes involved in this study as many are labelled as anti-cancer drugs, such as chalcones and stilbenes [35, 36]. This therefore leaves the question; with other mechanisms of action being highlighted within this piece of research, could these compounds classes be chemically re-engineered to have lower toxicity rates and be used in a variety of ways? Compounds that target bacterial growth, efflux pump activity and biofilm formation are of great importance for combating and eradicating bacterial infections from a broad range of species. Specific compounds present in this study exhibit all of these mechanisms.

This study has highlighted the importance of whole-cell phenotypic evaluation in identifying other possible mechanisms of actions for inhibitor molecules whilst indicating how the chemical classes of ketones, chalcones and stilbenes have the potential to help in the fight against antimicrobial resistance being novel efflux pump and biofilm inhibitors.
